# RNA-Seq combined with population-level analysis reveals important candidate genes related to seed size in flax (*Linum usitatissimum* L.)

**DOI:** 10.3389/fpls.2022.1015399

**Published:** 2022-10-25

**Authors:** Haixia Jiang, Dongliang Guo, Yuanyuan Liu, Leilei Zhu, Fang Xie, Liqiong Xie

**Affiliations:** Xinjiang Key Laboratory of Biological Resources and Genetic Engineering, College of Life Science and Technology, Xinjiang University, Urumqi, China

**Keywords:** seed size, flax (*Linum usitatissimum* L.), transcriptome sequencing, population-level analysis, nucleotide diversity

## Abstract

Seed size is a key determinant of crop yields. Understanding the regulatory mechanisms of seed size is beneficial for improving flax seed yield. In this study, the development of large flax seeds lagged behind that of small seeds, and 1,751 protein-coding genes were differentially expressed in early seeds, torpedo-stage embryos, and endosperms of CIli2719 and Z11637 using RNA sequencing. Homologous alignment revealed that 129 differentially expressed genes (DEGs) in flax were homologous with 71 known seed size–related genes in *Arabidopsis thaliana* and rice (*Oryza sativa* L.). These DEGs controlled seed size through multiple processes and factors, among which phytohormone pathways and transcription factors were the most important. Moreover, 54 DEGs were found to be associated with seed size and weight in a DEG-based association study. Nucleotide diversity (π) analysis of seed size–related candidate DEGs by homologous alignment and association analysis showed that the π values decreased significantly during flax acclimation from oil to fiber flax, suggesting that some seed size–related candidate genes were selected in this acclimation process. These results provide important resources and genetic foundation for further research on seed size regulation and seed improvement in flax.

## Introduction

Seed size is a key determinant that affects crop yield and seed quality (Guo et al., 2020; Hu et al., 2021). Seed size has undergone strong selection pressure under natural and artificial selection during domestication. Seedless or small-seeded watermelons are used for flesh consumption, whereas edible-seeded watermelons are used for seed consumption and usually have larger seeds (Gong et al., 2022). Fiber flax (used to obtain stem fiber) usually has small seeds, whereas oil flax (used to produce seed oil) has large seeds (Guo et al., 2020). Although the seed size of different subgroups in the same species is significantly diverse, the molecular mechanisms of morphological divergence and selection features for seed size are still lacking.

The seeds of dicotyledons are composed of a seed coat, an endosperm, and an embryo. The embryo is a diploid zygote formed by the sperm–egg cell fusion during double fertilization. Embryo development starts with the zygote, which undergoes proembryo and embryo differentiation and finally develops a mature embryo instead of the endosperm to fill the seeds (Sundaresan, 2005; Ren et al., 2019). The endosperm is a triploid tissue formed by the fusion of another sperm cell with two polar nuclei of the central cell (Lafon-Placette and Köhler, 2014). Endosperms provide nutrients and signal sources for embryo development and are closely related to seed size. Endosperm development is divided into syncytial, cellularization, differentiation, and death phases (Orozco-Arroyo et al., 2015). Among these, endosperm cellularization is a key phase in determining seed size. Precocious endosperm cellularization produces small seeds, whereas delayed endosperm cellularization results in large seeds (Orozco-Arroyo et al., 2015), and failure of endosperm cellularization leads to embryo abortion (Hehenberger et al., 2012). The seed coat surrounding the developing embryo and endosperm is derived from ovule integuments and ultimately affects seed size (Garcia et al., 2005; Orozco-Arroyo et al., 2015). Therefore, the seed size is determined by the coordinated growth of the endosperm, embryo, and seed coat (Garcia et al., 2005).

In recent decades, multiple specific processes and factors in plants have been verified to control seed size. Phytohormones are biologically active compounds crucial for seed size regulation, and almost all known phytohormones signaling pathways are involved in seed size and weight, including auxin, brassinosteroids (BRs), abscisic acid (ABA), gibberellin, cytokinin (CK), jasmonate (JA), and ethylene (ET) signaling pathways (Li and Li, 2016; Li et al., 2019; Chen et al., 2021; Hu et al., 2021). In addition, transcription factors (TFs), photosynthetic product accumulation and transportation, epigenetics, G-protein signaling pathway, IKU (HAIKU) pathway, mitogen-activated protein kinase (MAPK) signaling pathway, ubiquitin–proteasome pathway, and maternal control factors also play important roles in controlling seed size and weight (Li and Li, 2014; Li and Li, 2015; Orozco-Arroyo et al., 2015; Li and Li, 2016; Li et al., 2018; Li et al., 2019; Cao et al., 2020; Chen et al., 2021; Li et al., 2022).

Cultivated flax is a famous crop grown in North America and some European countries, which was derived from pale flax (*Linum bienne*) and classified into two main cultivation types: oil flax and fiber flax (Cloutier et al., 2012; Guo et al., 2020; Jiang et al., 2021). In contrast to the one-way breeding goal of grain crops for seed yield, the opposite selection for stem and seed yields of flax has caused great differences in seed yield–related traits, particularly seed size. Compared with fiber flax, oil flax has significantly longer seed length, wider seed width, heavier seed weight, and higher seed production (Guo et al., 2020). To date, only five simple sequence repeat markers, 16 quantitative trait loci, 13 candidate genes, and 13 imprinted genes have been identified to be related to flax seed size and weight using a mixed linear model (MLM) and/or a general linear model (GLM) (Soto-Cerda et al., 2014; Xie et al., 2018a; Xie et al., 2018b; Guo et al., 2020; Jiang et al., 2021). However, whether the significant difference in seed size is caused by the variation in seed development between oil flax and fiber flax; what the regulatory factors of flax seed size are; and whether seed size, as a key trait, has experienced selection in the history of flax domestication are still unclear.

Herein, we performed morphological and cellular observations, RNA sequencing (RNA-Seq), homologous comparison, and population-level analysis to explore the influence of developmental differences in the endosperm, embryo, and seed coat on seed size; identify the regulatory genes of seed size; and analyze selective sweep signals of seed size–related candidate genes in flax. Morphological and cellular observations revealed that the development of small flax seeds occurred earlier than that of large seeds. The transcriptomic analysis of early seeds, torpedo-stage embryos and endosperms between the large-seed variety CIli2719 (C) and small-seed variety Z11637 (Z), identified 1,751 protein-coding differentially expressed genes (DEGs). Homologous comparison revealed 129 DEGs in flax to be homologous to seed size regulatory genes that were functionally verified in *Arabidopsis thaliana* and rice (*Oryza sativa* L.). Furthermore, 54 DEGs were identified as seed size– and weight-related candidate genes using population-level analysis. The nucleotide diversity (π) of candidate seed size–related DEGs identified both by homologous alignment and DEG-based association analysis in fiber flax was significantly lower than that of oil flax, suggesting that seed size–related candidate DEGs might experience artificial selection during flax domestication. These findings provide candidate genes and molecular mechanisms for further studies on seed size regulation and seed improvement in flax.

## Materials and methods

### Plant material and sample collection

The 200 flax varieties, namely, 78 oil, 71 oil-fiber, and 51 fiber flax, grown in Dali in 2016 (2016DL), Urumqi in 2017 (2017UR), Urumqi in 2019 (2019UR), and YiLi in 2019 (2019YL) were used for population-level analysis. The origin, planting, and seed size phenotypic measurements of 200 flax germplasms were performed as previously described (Guo et al., 2020). A large seed variety CIli2719 (C; ~10.5 g of 1,000-seed weight) and a small seed variety Z11637 (Z; ~3.7 g of 1,000-seed weight) with extreme seed size difference planted in the Miquan experiment filed, Urumqi, Xinjiang, were chosen for transcriptome sequencing and dynamic analysis of seed development. Embryo and endosperm samples that corresponded to the torpedo stages according to our previous study were collected 7 days after pollination (DAP) (Jiang et al., 2021). The seed samples of 2 DAP were collected from at least 50 seeds, frozen immediately in liquid nitrogen, and then stored at −80°C for RNA-Seq and real-time quantitative PCR (qRT-PCR) validation analysis. Three independent biological replicates were set up for all the samples.

### Morphological and cellular analysis

To study dynamic changes in seed development, 1–13 DAP seeds of C and Z were collected for morphological and cellular observations. The lengths of the outer integument, inner integument, and embryo of at least 30 developing seeds were measured using Image J 1.8.0 after their images were captured by a high-resolution scanner.

To observe whole-mount seeds, developing seeds were slit at both ends, fixed in an ethanol/acetic acid/formaldehyde/glycerol solution (FAA; 90 ml 50% ethanol, 5 ml acetic acid, 5 ml 38% formaldehyde, and 5 ml glycerol), and incubated at 4°C. The samples were then washed with 90% and 70% ethanol for 30 min each. A chloral hydrate/glycerol/water solution (2 ml glycerol, 4 ml water, and 8 g chloral hydrate) was used to clear the samples before being visualized by a stereomicroscope (Nikon SMZ25, Japan).

The developing seeds fixed in FAA were sliced into 6-μm serial sections with a microtome (Leica RM2235, Germany) after a series of ethanol gradient dehydration, dimethylbenzene transparent treatment, and paraffin penetration and embedding. The sections were then counterstained with 1% safranin and 1% fast green (Pan et al., 2008). The images were observed using an optical microscope (Nikon Eclipse E200, Japan), and photographs were acquired using a microimage system software.

### RNA extraction, library construction, sequencing, read mapping, and gene expression analysis

RNA-Seq of 7 DAP embryo and endosperm and 2 DAP seed samples was performed using the Illumina NovaSeq6000 platform at Novogene Bioinformatics Institute, Beijing, China, as previously described (Jiang et al., 2021). Eventually, 858.68 million raw reads and 826.98 million clean reads were acquired from 18 libraries. Pearson’s correlation coefficients (*r*) between biological replicates were calculated with the normalized expression levels of log10(FPKM + 1) (FPKM, fragments per kilobase of exon model per million mapped fragments).

### Identification and functional annotation of differentially expressed genes

The differential expression analysis of the embryo, endosperm, and seeds between CIli2719 and Z11637 was performed using the DESeq2 R package (Love et al., 2014). The *p*-values were adjusted using Benjamini and Hochberg’s approach for controlling the false discovery rate (FDR/*p*_adj_). Genes with FDR < 0.01 and |log2(fold change)| > 1 were assigned as significant DEGs. The Venn diagrams of DEGs from the six samples were drawn with TBtools (Chen et al., 2020a). The heat map and hierarchical clustering of DEGs were generated using TBtools software with FPKM (Chen et al., 2020a). Gene Ontology (GO) enrichment and Kyoto Encyclopedia of Genes and Genomes (KEGG) pathway analysis of DEGs were based on all expressed background genes and performed using the GOseq R package (Young et al., 2010) and KOBAS software (Chen et al., 2020b). GO and KEGG terms with *p*_adj_ < 0.05 and *p* < 0.05 were considered significantly enriched by DEGs, respectively. Differential expression, GO enrichment, and KEGG enrichment analyses are all proceeded on the cloud platform NovoMagic (https://magic.novogene.com/customer/main#/login).

### Real-time quantitative PCR analysis

After total RNA extraction, quantification, qualification, and first-strand cDNA synthesis, qRT-PCR analysis of 28 genes was performed using an abm EvaGreen Express 2× qPCR MasterMix and a BioRad^®^ CFX96 Real-Time PCR System with three biological replicates. The thermal cycler program was as follows: 95°C for 30 s, 40 cycles at 95°C for 5 s, 60°C for 15 s, followed by a melting curve program with an increase from 65°C to 95°C in 5-s increments of 0.5°C. The relative expression levels of genes were quantified by the geometric mean of the housekeeping genes *ETIFI* (eukaryotic translation initiation factor 1), *GAPDH* (glyceraldehyde 3-phosphate dehydrogenase), and *ETIF5A* (eukaryotic translation initiation factor 5A) (Huis et al., 2010; Hobson and Deyholos, 2013; Jiang et al., 2021) and analyzed with the **2^−^** ^△△^ **^Ct^** method (Livak and Schmittgen, 2001). The primers used for the qRT-PCR analysis are shown in **Supplementary Table S1**.

### Obtaining flax seed size–related genes by homologous analysis

The seed size–related genes (verified by functional analysis) of *Arabidopsis* (dicotyledon) and rice (monocotyledon) were obtained from the literature and databases, including The Arabidopsis Information Resource (https://www.arabidopsis.org/), the Arabidopsis Biological Resource Center (https://abrc.osu.edu/researchers), the Nottingham Arabidopsis Stock Centre (http://arabidopsis.info/BasicForm), the RIKEN BioResource Research Center (https://epd.brc.riken.jp/en/), and the China Rice Data Center (https://www.ricedata.cn/) (Guo et al., 2021). Peptide sequences of known seed size–related genes in *Arabidopsis* and rice were acquired from the Phytozome v13 database (https://phytozome-next.jgi.doe.gov/). Subsequently, these peptide sequences were aligned to the flax genome (*Linum usitatissimum* v1.0), and flax genes with high-scoring (*E*-value < 1E−50) blast hits were obtained. If these genes were DEGs, they were considered flax seed size–related candidate genes, and the known seed size–related genes of *Arabidopsis* and rice with the lowest *E*-value were defined as the homologous genes of seed size–related candidate DEGs. A Venn diagram was drawn with TBtools (Chen et al., 2020a).

### Candidate gene–based association study of flax differentially expressed genes

A DEG-based association study of seed length, seed width, and 1,000-seed weight was performed with GLM and MLM using the TASSEL 5.0 software (Bradbury et al., 2007). The 12,753 single-nuclear polymorphisms (SNPs) in 1,751 DEGs and seed size–related traits of 200 flax varieties were obtained from our previous study (Guo et al., 2020). Both GLM and MLM analyses were performed as our previous description (Jiang et al., 2021), and the significance threshold was determined to be 0.05/n (n represents total SNPs, −log_10_(*P*) = 5.41) for GLM and 1/n (−log_10_(*P*) = 4.11) for MLM. DEGs detected repeatedly in at least two environments were considered candidate genes relevant to seed size.

### Identification of selection signatures of seed size–related candidate differentially expressed genes

To detect the genetic variation in 54 and 129 seed size–related candidate DEGs obtained by DEG-based association studies (AS-DEGs) and peptide sequence alignments (PSA-DEGs) in two major morphological types of cultivated flax, oil flax (78 germplasms) and fiber flax (51 germplasms) subgroups, 894 (54 AS-DEGs), 902 (129 PSA-DEGs), and 129 (housekeeping genes) SNPs (Jiang et al., 2021) were acquired from our previously constructed high-density genotype map (Guo et al., 2020). The π values of seed size–related candidate DEGs between the two flax subgroups were computed at the gene and SNP levels, as described in our previous study (Jiang et al., 2021).

### Statistical analysis

Statistical analysis of the test data was performed using GraphPad Prism 8.0.2 (263) software. Phenotypic difference analysis and nucleotide diversity analysis were performed with unpaired two-tailed *t*-tests.

## Results

### Seed development characteristics in Z11637 and CIli2719

**Figure 1:**
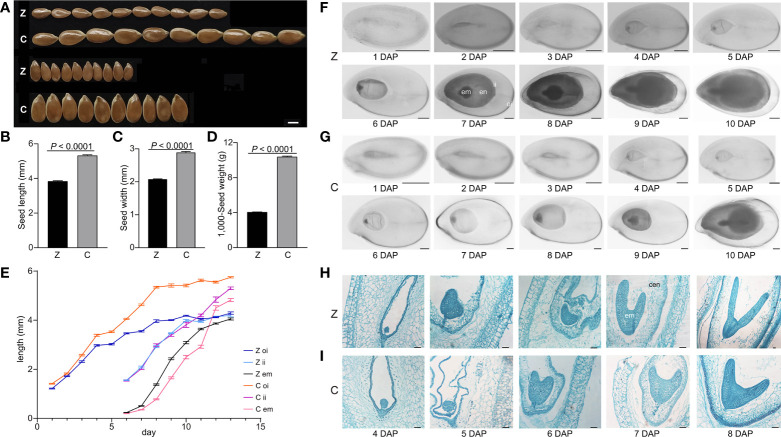
Analyses of seed development in Z and C. **(A)** Seed phenotypes of Z and C. **(B–D)** Seed length **(B)**, seed width **(C)**, and 1,000-seed weight **(D)** of Z and C. *p*-values were analyzed by two-tailed *t*-test. **(E)** Outer integument length, inner integument length, and embryo length (mm) of Z and C. **(F, G)** Morphology of developing flaxseed of Z **(F)** and C **(G)**. **(H, I)** Microscopy images of developing flaxseed of Z **(H)** and C **(I)**. Z, Z11637; C, CIli2719; oi, outer integument; ii, inner integument; en, endosperm; em, embryo; cen, cellularized endosperm. Scale bars, 2 mm **(A)**, 500 µm **(F, G)**, and 50 µm **(H, I)**.

To explore the differences between small and large seeds, a small seed variety Z and a large seed variety C with extreme differences (*p* < 0.0001) in seed length, seed width, and 1,000-seed weight (**Figures 1A–D**) were chosen for seed dynamic development analysis. We measured the lengths of outer integument, inner integument, and embryo during seed development. The outer integument grew rapidly in the early stage (1–4 DAP) of seed development, and the length of C was significantly longer than that of Z. The size difference in the inner integument and embryo was not obvious between C and Z, which indicates that the outer integument is the main factor determining seed size in the early stage of seed development. There was no significant difference in inner integument length before 10 DAP. After 10 DAP, the inner integument of C continued to grow rapidly and exceeded the length of Z, whereas the inner integument Z grew slowly and reached the inner edge of the outer integument. Embryos grew rapidly at 7–11 DAP, and the size of embryos in C was significantly smaller than that in Z. Subsequently, the embryo development of Z gradually slowed and reached the maximum; however, C continued to grow rapidly and exceeded the embryo size of Z. At 13 DAP, seed development in Z was completed, whereas seed development in C continued (**Figure 1E**). These results indicated that the seed development of C lagged behind that of Z.

To further explore the seed development process of Z and C, morphological and cellular observations were performed. The results showed that the seed development of C lagged behind that of Z after fertilization. The Z and C seeds were both in the globular embryo stage at 4 DAP. The seeds of Z reached the heart embryo stage and formed cellularized endosperm, whereas the C seeds were still in the globular embryo stage at 5 DAP (**Figures 1F–I**). At 6 DAP, the Z seeds developed from the heart to the early torpedo stage, in which the embryo and the endosperm progressively grew, whereas the C seeds were in the heart stage. At 7 DAP, the Z seeds were in the late torpedo embryo stage, and the endosperm grew in size; however, the C seeds developed into early torpedo. At 8 DAP, the Z seeds had developed into a cotyledon embryo stage, during which the endosperm began to degenerate, and the C seeds were still in the late torpedo stage (**Figures 1F–I**). At the late cotyledon stage, the embryo occupied the whole embryo sac with a thin endosperm remaining in the mature seeds (**Figures 1F, G**). These findings showed that the development of the embryo, endosperm, and seed coat of Z was significantly earlier than that of C.

### Generation of RNA-Seq data set from flax seeds

To analyze the developmental differences in the embryo, endosperm, and seeds between Z and C, the embryo and endosperm at 7 DAP and seeds at 2 DAP were constructed to perform high-throughput RNA-Seq using the Illumina NovaSeq6000 platform. A total of 858.68 million, with an average of 47.70 million, 150-bp paired-end raw reads were acquired from 18 libraries, ranging from 40.72 million to 57.37 million reads from each library. After removing low-quality sequencing reads from raw data, 826.98 million (124.06 Gb) clean reads were obtained from 18 libraries. The ranges of Q20 and Q30 percentages and GC content for the 18 libraries were 97.41%–98.54%, 93.19%–95.28%, and 47.53%–49.32%, respectively. Meanwhile, 37.29–52.58 million (91.21%–96.62%) clean reads from 18 libraries were mapped to the flax reference genome. The multiple and uniquely mapping ratio of all libraries were 3.05%–4.70% and 86.81%–92.80%, respectively (**Supplementary Table S2**). Pearson correlation coefficients between any two of the three biological replicates for each sample were higher than 0.945 (**Supplementary Figure S1** and **Table S3**), indicating that the RNA-Seq data were highly reliable and reproducible.

### Identification of differentially expressed genes

**Figure 2:**
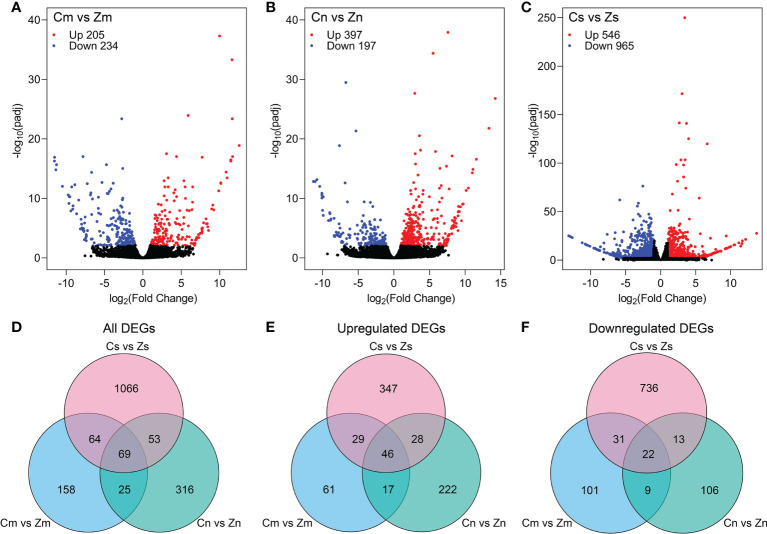
Identification of differentially expressed genes (DEGs) in torpedo-stage embryo and endosperm, and early seeds between C and Z. **(A–C)** Volcano plots showing DEGs in the embryo **(A)**, endosperm **(B)**, and seeds **(C)** between C and Z. **(D–F)** All **(D)**, upregulated **(E)**, and downregulated **(F)** DEGs in the embryo, endosperm, and seeds comparisons.

To study the differences at transcription level among the embryo, endosperm, and seeds of Z and C, differential gene expression analysis was performed using the DESeq2 R package (Love et al., 2014) with the criteria of |log2(fold change)| > 1 and *p*_adj_ < 0.01. In total, 439 (205 upregulated and 234 downregulated), 594 (397 upregulated and 197 downregulated), and 1,511 (546 upregulated and 965 downregulated) DEGs were identified in the embryo (Cm vs. Zm), endosperm (Cn vs. Zn), and seeds (Cs vs. Zs), respectively (**Figures 2A–C**). Among these DEGs, 316 (153 upregulated and 163 downregulated), 463 (313 upregulated and 150 downregulated), and 1,252 (450 upregulated and 802 downregulated) genes were protein-coding genes in the embryo, endosperm, and seeds, respectively (**Figures 2D–F**). A total of 1,751 protein-coding DEGs were identified in three comparisons (**Figure 2D**). These results indicated that hundreds of genes were differentially expressed in Z and C.

### Validation of transcriptome data

To validate the RNA-Seq results, 28 genes, including 5 genes in the embryo, 12 genes in the endosperm, and 11 genes in seeds, were randomly selected for qRT-PCR analysis. The transcriptional levels of these tested genes through qRT-PCR analysis showed a trend similar to that of the RNA-Seq data (**Supplementary Figure S2**), indicating the high reliability of the RNA-Seq data.

### Functional analysis of differentially expressed genes

To classify the functions of flax DEGs, GO analysis was performed for DEGs in the embryo, endosperm, and seeds. As a result, 20 DEGs identified in the endosperm were significantly enriched into 14 GO terms (*p*_adj_ *<* 0.05), including five biological processes, five cellular components, and four molecular function categories. In the biological process category, DEGs were significantly enriched in cellular carbohydrate, cellular glucan, glucan, cellular polysaccharide, and polysaccharide metabolic processes. In the cellular component category, the significantly enriched terms were extracellular region, apoplast, cell wall, external encapsulating structure, and cell periphery. In the molecular function category, glucosyltransferase activity, xyloglucan:xyloglucosyl transferase activity, phosphoenolpyruvate carboxykinase activity, and sucrose synthase activity were identified (**Supplementary Figure S3A** and **Table S4**). In the seeds, 27 DEGs were significantly enriched in ADP binding and chaperone binding, according to molecular function categories (**Supplementary Figure S3B** and **Table S4**). However, the DEGs identified in the embryo were not significantly enriched by any GO strategy (**Supplementary Figure S3C**).

KEGG analysis was used to further investigate the functions of DEGs. Eight DEGs identified in the embryo were significantly enriched in four pathways (*p* < 0.05), namely, plant–pathogen interaction, starch and sucrose metabolism, phosphatidylinositol signaling system, and inositol phosphate metabolism. Twelve endosperm DEGs were significantly enriched in three pathways (*p* < 0.05), namely, peroxisome, starch and sucrose metabolism, and carbon metabolism, and 31 DEGs identified in 2 DAP seeds were significantly enriched in five pathways (*p* < 0.05), namely, starch and sucrose metabolism, protein processing in the endoplasmic reticulum, phenylpropanoid biosynthesis, histidine metabolism, and plant hormone signal transduction (**Supplementary Table S5**). The DEGs identified in the embryo, endosperm, and seeds were significantly enriched in starch and sucrose metabolism, indicating that carbohydrate utilization played a key role in flax seed development, although the mature seeds were full of oil. Additionally, the difference in sugar metabolism affected seed size.

### Important candidate genes related to seed size in flax

To identify potential seed size–related genes from the 1,751 DEGs, known seed size–related genes of *Arabidopsis*, rice, and flax were identified from previous literature and public databases. In total, 156, 224, and 1 seed size–related regulatory genes that have been functionally verified were obtained from *Arabidopsis*, rice, and flax, respectively (**Supplementary Table S6**). Peptide sequence alignment revealed 1,413 and 1,685 flax genes were homologous to Arabidopsis and rice seed size-related genes, of which 76 and 95 genes were DEGs, respectively (**Supplementary Figure S4** and **Tables S7**, **S8**). Among these DEGs, 34 and 53 genes were the only homologous genes in *Arabidopsis* and rice, respectively, and 42 were common homologous genes in both (**Supplementary Figure S4**). In total, 129 DEGs were identified to be homologous with 71 (36 *Arabidopsis* and 35 rice) known seed size–related genes (**Supplementary Table S8**). Analysis of the expression patterns of 129 DEGs in various tissues revealed that most genes were preferentially expressed in flax seed tissues (**Supplementary Figure S5**), suggesting that these DEGs play a role in seed development. Further analysis revealed that 129 DEGs regulated seed size and weight *via* multiple processes and factors.

**Figure 3:**
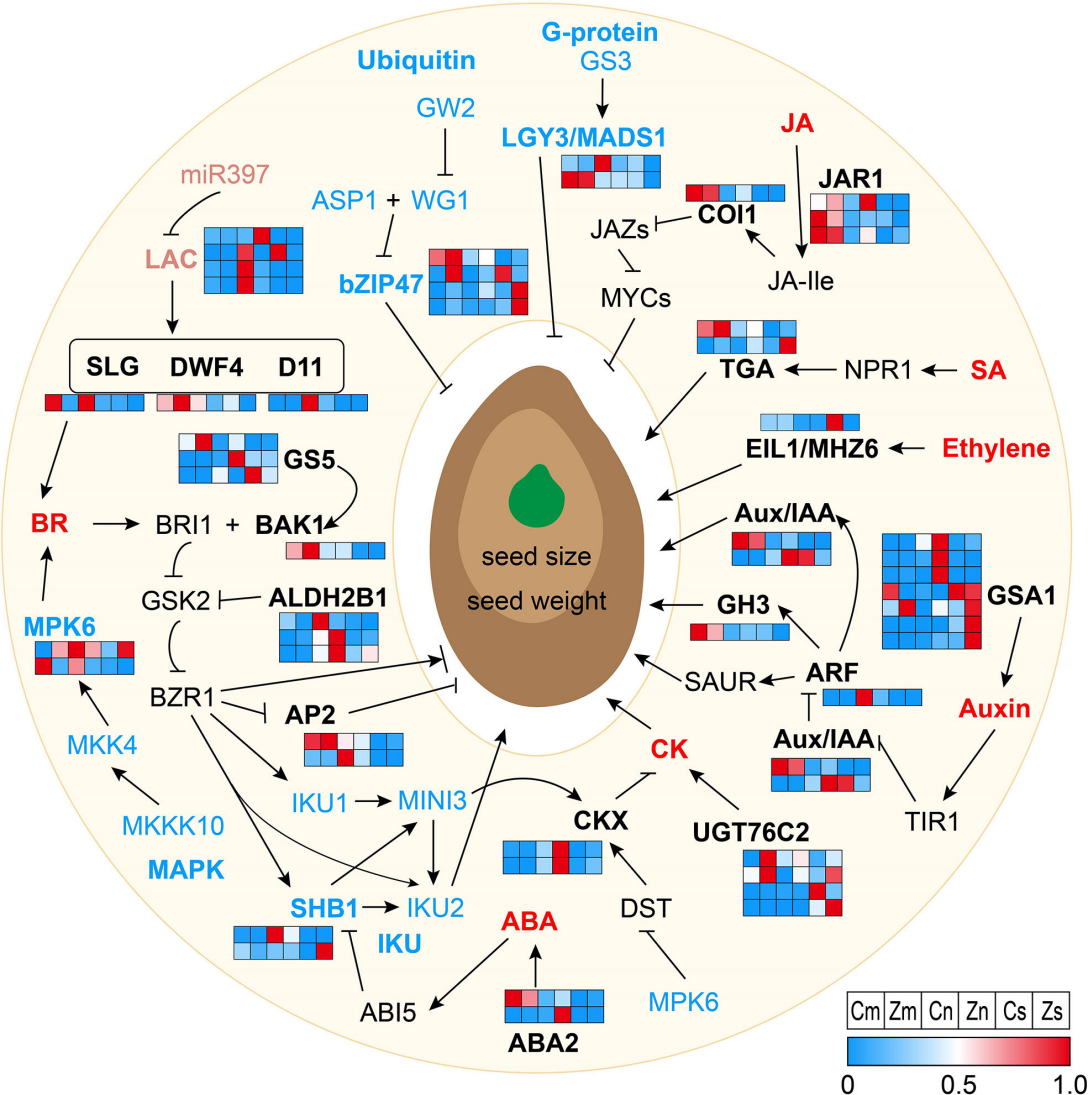
Regulation network *via* multiple signaling pathways for seed size and weight. Normal arrows represent activated or positive regulation, and block arrows indicate inhibition or negative regulation. Different color letters show proteins that are involved in different signaling pathways. Black letters represent the phytohormone pathways; blue letters represent the G-protein, ubiquitin–proteasome, mitogen-activated protein kinase (MAPK), and IKU pathways, and pink letters represent the microRNA regulators. Red letters indicate the phytohormones. Black, blue, and pink bold letters in the pathways represent the differentially expressed genes (DEGs) identified in this study. Heatmaps of DEGs participating in signaling pathways are displayed based on the normalized FPKM values. FPKM, fragments per kilobase of exon model per million mapped fragments.

Seed size and weight were regulated by DEGs through phytohormone pathways. According to KEGG and the existing literature, 39 of 129 DEGs were involved in phytohormone pathways, including the auxin, BR, ABA, CK, JA, SA (Salicylic acid), and ET signaling pathways (**Figure 3** and **Supplementary Table S9**). In the auxin signaling pathway, the genes encoding the auxin response factor ARF, the auxin influx carrier AUX3, the auxin-responsive protein IAA3, and the auxin-responsive protein GH3 were upregulated in Cn or Cs compared with those in Zn or Zs, respectively. In addition, compared with Zs, Zn, or Zm, six *GSA1* (UDP-glucosyltransferase) were downregulated in Cs or Cn, and one *GSA1* was upregulated in Cm. In the BR pathway, one *AP2* (DNA-binding protein), one *ALDH2B1* (aldehyde dehydrogenase), one *D11* (cytochrome P450 protein), one *DWF4* (cytochrome P450 protein), one *GS5* (serine carboxypeptidase), one *BAK1* (BRI1-associated receptor kinase), and one *SLG* (BAHD acyltransferase) were upregulated in Cs, Cm, and/or Cn, whereas the other five genes, namely, one *AP2*, two *ALDH2B1*, and two *GS5*, were downregulated in Cs, Cm, and/or Cn. In the CK pathway, except for one *UGT76C2* (UDP-glucosyltransferase) that was upregulated in Cs, the other three *UGT76C2* and two *CKX* (cytokinin oxidase) genes were downregulated in Cs or Cm. In the JA pathway, one orthologue of *COI1* (JA receptor) and two orthologues of *JAR1* (jasmonate-amido synthetase) were downregulated in Cn or Cs; however, another *JAR1* gene was upregulated in Cs. Six genes involved in ABA (four genes) and SA (two genes) signaling pathways were significantly downregulated in Cs or Cn, and the gene encoding ET-insensitive protein 3 related to the ET pathway was upregulated in Cs (**Figure 3** and **Supplementary Table S9**). Our results illustrated that complex signaling pathways vary dynamically in seed tissues of different sizes.

TFs are important factors that control seed size. In this study, 18 TF DEGs, namely, MADS (4), NAC (4), bZIP (4), AP2 (2), ARF (1), EIL (1), GRAS (1), and WRKY (1), were found to be related to flax seed size (**Supplementary Table S10**). Among these, 14 (7 upregulated and 7 downregulated) and 3 (3 upregulated) TFs were specifically differentially expressed in Cs versus Zs and Cn versus Zn, respectively. Moreover, the NAC TF *Lus10002083*, an orthologue of *OsNAP*, was differentially expressed in both Cs versus Zs and Cm versus Zm.

Photosynthetic product accumulation and transportation processes are associated with seed size and weight. Nine (two upregulated and seven downregulated), three (one upregulated and two downregulated), and six (four upregulated and two downregulated) genes were differentially expressed in Cs versus Zs, Cm versus Zm, and Cn versus Zn, respectively. Among these, two genes (one upregulated and one downregulated) were differentially expressed in all three comparisons (**Supplementary Table S11**).

Epigenetic, G-protein signaling, IKU, MAPK signaling, ubiquitin–proteasome, and maternal control processes regulated seed size and weight. Three chromatin modifications and one microRNA gene were upregulated in Cs versus Zs. Two chromatin modification genes (one upregulated and one downregulated) were differentially expressed in Cm versus Zm. One chromatin modification gene and three microRNA genes were differentially expressed in Cn versus Zn (**Supplementary Table S12**). Additionally, there were 2 G-protein signaling pathway, 2 IKU pathway, 11 MAPK signaling pathway, 4 ubiquitin–proteasome pathway, and 9 maternal control genes that showed differential expression in Cs versus Zs, Cm versus Zm, or Cn versus Zn (**Figure 3** and **Supplementary Table S13**). These results suggest that these DEGs regulate flax seed development and seed size through multiple regulatory processes and factors.

### Differentially expressed gene–based association study for three flax seed size–related traits

**Figure 4:**
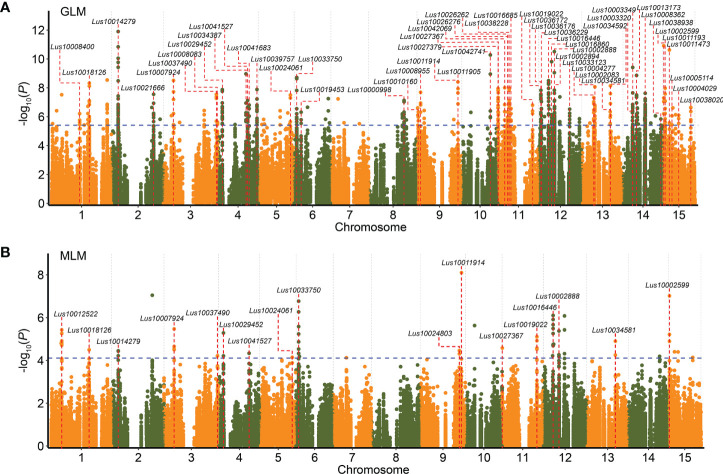
Differentially expressed gene (DEG)–based association study for seed size and weight. **(A, B)** The overlapping Manhattan plots for seed length, seed width, and 1,000-seed weight in four environments (including 2016DL, 2017UR, 2019UR, and 2019YL) using general linear model (GLM) **(A)** and mixed linear model (MLM) **(B)**. Blue dashed lines represent significance thresholds (−log_10_(*P*) = 5.41 in **A**; −log_10_(*P*) = 4.11 in **B**). Red dashed lines indicate DEGs that were repeatedly detected in at least two environments related to seed size and weight.

**Figure 5:**
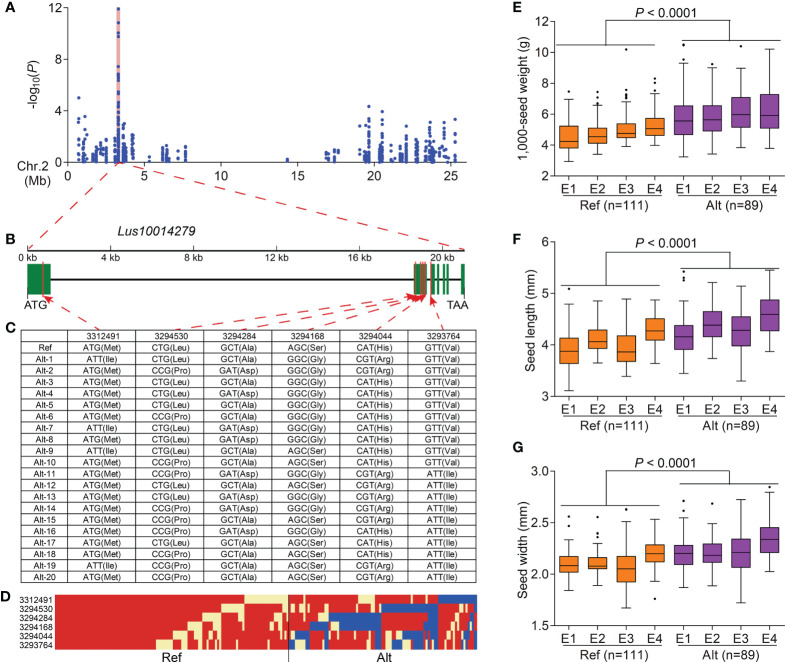
Identification of a seed size–related candidate gene on chromosome 2. **(A)** Local Manhattan plots for 1,000-seed weight in 2016DL on chromosome 2, and a shaded pink column highlights the position of *Lus10014279*. **(B)** Gene structure of *Lus10014279*. Green rectangles and black line represent exons and introns, respectively. **(C)** DNA polymorphism in *Lus10014279*. **(D)** Schematic diagram of the structural variation in *Lus10014279.* **(E–G)** Box plots for 1,000-seed weight **(E)**, seed length **(F)**, and seed width **(G)** based on the haplotypes for *Lus10014279* in 2016DL (E1), 2017UR (E2), 2019UR (E3), and 2019YL (E4). The difference was analyzed by two-tailed *t*-tests.

To further study the associations between DEGs and flax seed size traits, 1,751 DEGs were used to conduct a DEG-based association study of three traits (seed length, seed width, and 1,000-seed weight) related to flax seed size using GLM and MLM in TASSEL 5.0 (Bradbury et al., 2007). A total of 112 DEGs, containing 271 significant association peaks, were related to the three traits. Among these DEGs, 54 DEGs (including 186 SNPs) were repeatedly observed in at least two environments (**Figures 4A, B** and **Supplementary Table S14**). A significant signal peaks on chromosome 2 were repeatedly detected in 2016DL, 2017UR, 2019UR, and 2019YL, containing 10 repetitive SNPs located in *Lus10014279*, encoding a pentatricopeptide repeat superfamily protein (**Figure 5A** and **Supplementary Table S14**). There were 75 SNPs included in this gene, among which six SNPs caused nonsynonymous mutations to produce 21 haplotypes, which were classified into haplotype reference (Ref) and haplotype alternate (Alt) groups (**Figures 5B–D**). The results showed that flax varieties with haplotype Alt had significantly larger 1,000-seed weight, longer seed length, and wider seed width than those in haplotype Ref (**Figures 5E–G**). Overall, these findings suggested that the DEG *Lus10014279* is a reliable candidate gene that participates in seed size regulation in flax.

### Selective sweep signals in seed size–related candidate differentially expressed genes

**Figure 6:**
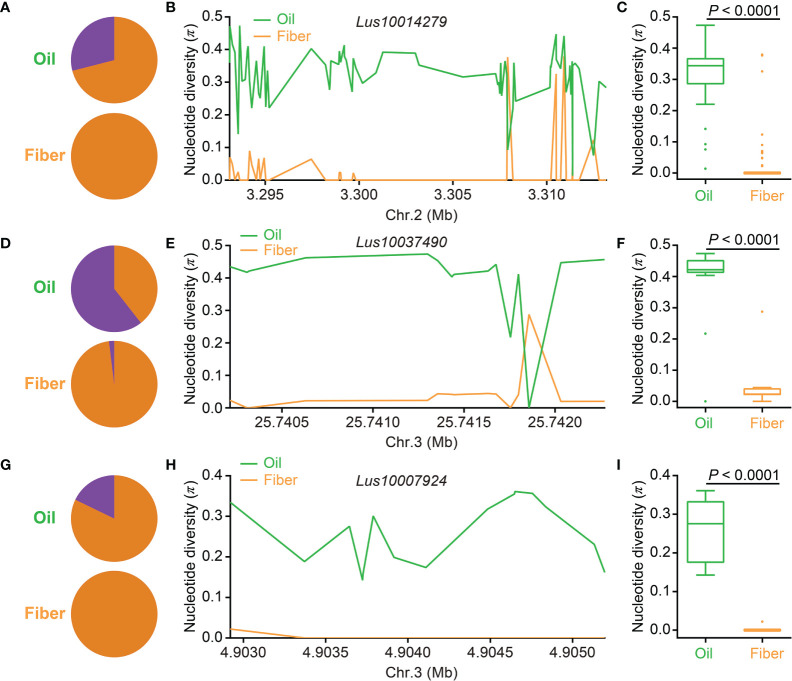
Distribution of nucleotide diversity (π) and allele frequency in three seed size–related candidate differentially expressed genes (DEGs) identified by a DEG-based association study between the oil flax and fiber flax subgroups. **(A, D, G)** Allele frequency distribution of single-nucleotide polymorphisms (SNPs) located in *Lus10014279* **(A)**, *Lus10037490* **(D)**, and *Lus10007924* **(G)** in the oil flax (green) and fiber flax (orange) subgroups. The proportions of large-seed and small-seed alleles are indicated by purple and orange colors, respectively. **(B, E, H)** Distribution of the π of *Lus10014279* on chromosome 2 **(B)**, *Lus10037490* on chromosome 3 **(E)**, and *Lus10007924* **(H)** on chromosome 3 between the oil flax and fiber flax subgroups. **(C, F, I)** Box plots for the π of *Lus10014279* **(C)**, *Lus10037490* **(F)**, and *Lus10007924* **(I)** in the oil flax and fiber flax subgroups. The difference was analyzed by two-tailed *t*-tests. Chr., chromosome.

Previous studies revealed a significant difference in seed size and weight between oil flax and fiber flax (*p* < 0.0001) and that some candidate genes related to flax seed size underwent artificial selection (You et al., 2017; Guo et al., 2020; Jiang et al., 2021). To study whether seed size experienced human selection in the flax domestication process, 894 (54 AS-DEGs) SNPs and 902 (129 PSA-DEGs) SNPs were collated to investigate nucleotide diversity in the oil flax and fiber flax subgroups. Compared with fiber flax, the π values of 54 AS-DEGs and 129 PSA-DEGs were much higher in the oil flax subgroup (*p* < 0.0001, *t*-test); however, the π values of housekeeping genes were similar in the two subgroups (**Supplementary Figure S6A**). Three AS-DEGs, *Lus10014279*, *Lus10037490*, and *Lus10007924*, containing 75, 15, and 15 SNPs in their genomic sequences, respectively, were used for nucleotide diversity analysis between the two flax subgroups. In *Lus10014279*, the lead SNP (position 3298424) had two alleles, namely, the large-seed allele “G” and the small-seed allele “A.” The “G” allele mainly distributed in the oil flax (28.99%) and none in the fiber flax subgroup (**Figure 6A**). Compared with fiber flax, the π values of the SNPs in *Lus10014279* were much higher in the oil flax subgroup (**Figures 6B, C**). In the lead SNP of *Lus10037490* (position 25742032, C/G), 60.56% of the varieties contained the large-seed allele “C” in the oil flax subgroup; however, only 1.96% varieties contained the “C” allele in the fiber flax subgroup (**Figure 6D**). In addition, the large-seed allele “C” of the lead SNPs in *Lus10007924* (position 4903371, C/T) mainly existed in the oil flax subgroup (17.81%), but not in the fiber flax subgroup (**Figure 6G**). Compared with fiber flax, the SNPs in *Lus10037490* (**Figures 6E, F**) and *Lus10007924* (**Figures 6H, I**) had higher nucleotide diversity in oil flax. Additionally, nucleotide diversity analysis of the other 11 AS-DEGs (**Supplementary Figures S6B–L**) and 14 PSA-DEGs (**Supplementary Figure S7**) demonstrated that their π values decreased significantly from oil flax to fiber flax. These results indicated that some seed size–related candidate DEGs might have undergone human selection during the domestication process from oil to fiber flax.

## Discussion

Seed size is an important agronomic trait for crop yield and is coordinately controlled by the endosperm, embryo, and seed coat growth (Berger et al., 2006; Li and Li, 2015; Li and Li, 2016; Guo et al., 2020). Herein, it was noted that the outer integument from the ovule integuments first provided a seed cavity for flax seed development, followed by the synergistic growth of the seed coat, endosperm, and embryo to complete the flax seed formation (**Figure 1**). There are three reasons for the large seed line C having larger seeds than the small seed line Z: (i) C had a larger seed coat than Z, which provided more cavities for the endosperm and embryo growth and set a larger upper limit to the final seed size of C (**Figure 1**); (ii) compared with Z, the timing of the endosperm cellularization of C was delayed (**Figures 1H, I**), which might be related to the large seed phenotype. This finding is consistent with previous reports that delayed endosperm cellularization results in large seeds, whereas precocious endosperm cellularization leads to small seeds (Garcia et al., 2003; Orozco-Arroyo et al., 2015); (iii) C underwent longer periods of embryo development and produced larger embryos than that of Z, thereby increasing seed size (**Figures 1E–G**), which was similar to a previous report in *Arabidopsis* (Ohto et al., 2009).

To analyze the differences in seed development and seed size between C and Z, we carried out three experiments, including RNA-Seq analysis, peptide sequence alignment, and DEG-based association study. RNA-Seq analysis of early-stage seeds and torpedo-stage embryos and the endosperm of C and Z, which had morphological differences in the size (**Figure 1E**), revealed 1,511, 439, and 594 DEGs in the seeds (Cs vs. Zs), embryo (Cm vs. Zm), and endosperm (Cn vs. Zn), respectively (**Figures 2A–C**). KEGG analysis showed that the DEGs at Cs versus Zs were significantly enriched in plant hormone signal transduction (**Supplementary Table S5**), indicating that phytohormones control early seed development. In addition, the DEGs identified in the three comparisons were significantly enriched in starch and sucrose metabolism (**Supplementary Table S5**), suggesting a vital role in flax seed development and seed size regulation.

To date, hundreds of genes involved in phytohormone pathways, TFs, photosynthetic product accumulation and transportation, epigenetics, G-protein, IKU, MAPK, ubiquitin–proteasome, and maternal control processes or factors have been identified and verified to control seed size (Chen et al., 2021; Li et al., 2022). Peptide sequence alignment indicated that 129 DEGs were homologous to 71 known regulatory genes, which correlated with the seed size in *Arabidopsis* and rice (**Supplementary Table S8**). Among these, 90 (69.77%), 15 (11.63%), and 33 (25.58%) genes were differentially expressed in Cs versus Zs, Cm versus Zm, and Cn versus Zn, respectively (**Supplementary Table S8**), indicating that early seed development plays a key role in seed size regulation. Interestingly, we found 39 of 129 DEGs involved in phytohormone pathways (**Supplementary Table S9**), of which 11 DEGs were involved in the auxin pathway, including *ARF4*, *GSA1*, *AUX3*, *IAA3*, and *CH3*, whose mutations affected the seed size in *Arabidopsis* and rice (Hu et al., 2018; Zhang et al., 2018; Dong et al., 2020; Hu et al., 2021; Qiao et al., 2021). Twelve DEGs were BR pathway–related genes containing *SLG*, *DWF4*, *D11*, *GS5*, *BAK1*, *ALDH2B1*, and *AP2*, which control seed size in *Arabidopsis* and rice (Jofuku et al., 2005; Wu et al., 2008; Xu et al., 2015; Zhu et al., 2015; Feng et al., 2016; Yuan et al., 2017; Ke et al., 2020). These results revealed a critical role of seed size regulation in phytohormone pathways, particularly the auxin and BR pathways. Eighteen TF orthologues of known seed size–related genes (*AP2*, *EIL1*, *MADS1*, *MADS6*, *ONAC022*, *ARF4*, *bZIP47*, *NAP*, *WRKY6*, and *GRAS19*) (Ohto et al., 2009; Liang et al., 2014; Hong et al., 2016; Song et al., 2020; Chen et al., 2021; Hao et al., 2021) were differentially expressed in flax seeds, embryos, and/or endosperm (**Supplementary Table S10**). Moreover, 14 photosynthetic product accumulation and transportation, 10 epigenetics, 2 G-protein, 2 IKU, 11 MAPK, 4 ubiquitin–proteasome, and 9 maternal control genes were differentially expressed in seeds, embryos, and/or endosperm samples (**Supplementary Tables S11**-**S13**). In summary, our findings showed that flax seed size is regulated by multiple regulatory processes, and the regulation processes of seed size are conservative in different species.

Additionally, with a DEG-based association study of three seed size traits in 200 flax natural populations, 54 DEGs correlated with seed size and weight (**Figure 4** and **Supplementary Table S14**). Among the 54 candidate genes, the gene *Lus10014279* displayed heavier 1,000-seed weight, longer seed length, and wider seed width in haplotype Alt than in haplotype Ref (**Figures 5E–G**). Moreover, three candidate genes were orthologues of two known regulatory genes that correlated with seed size in *Arabidopsis* and rice (**Supplementary Table S8**). *Lus10002083*, which encodes a plant-specific NAC transcriptional activator, located on chromosome 13, is an orthologue of *OsNAP* (**Supplementary Tables S8** and **S14**). Knockout of *OsNAP* significantly prolonged the grain-filling period and increased the 1,000-grain weight of rice (Liang et al., 2014). Two other candidate genes, *Lus10004277* and *Lus10011193*, are the orthologues of *AtLecRK-VIII.2* (**Supplementary Table S8**). *AtLecRK-VIII.2*, a positive regulator, controls seed size by regulating cell proliferation and differentiation of the seed coat in the MAPK signaling pathway (Xiao et al., 2021). Overall, these results indicate that these DEGs are credible and important candidate genes for regulating flax seed size.

Seed size and weight are among the primary factors influencing crop yield, and they usually undergo human selection during evolution (Lu et al., 2013; Hirsch et al., 2014; Zhou et al., 2015; Guo et al., 2020). In this analysis, compared with the oil flax subgroup, the nucleotide diversity of seed size and weight candidate DEGs identified both by a DEG-based association study and peptide sequence alignment was significantly decreased in the fiber flax subgroup (**Figure 6** and **Supplementary Figures S6** and **S7**), indicating that these candidate DEGs had undergone artificial selection in the flax domestication process. Interestingly, in crops, such as soybean, maize, and rice, large-seeded genotypes are usually selected during domestication (Lu et al., 2013; Hirsch et al., 2014; Zhou et al., 2015). However, in this study (**Figures 6A, D, and G**) and our previous study (Guo et al., 2020), the large-seeded genotypes were mainly distributed in the oil flax subgroup, with only a few or none in the fiber flax subgroup. Moreover, seed yield and 1,000-seed weight were negatively correlated with plant height in flax (Soto-Cerda et al., 2014). Purportedly, to maintain the dynamic balance between vegetative growth and reproductive growth, fiber flax reduces reproductive growth products (seeds) to increase vegetative growth products (flax fiber). In many crops, excessive vegetative growth may inhibit reproductive growth and reduce seed yield (Asano et al., 2011; Melchinger et al., 2016); however, the synergetic effect between vegetative and reproductive growth on population evolution is unknown. Herein, the small-seeded genotypes may have been selected along with the artificial selection of plant height during flax domestication from oil to fiber. Therefore, we propose that the interdependence and competition growth between seeds and fiber triggers flax to evolve into different subgroups.

## Conclusion

Seed size and weight are key factors that affect crop yield. In this study, the results showed that seed size is determined by the endosperm, embryo, and seed coat growth. Peptide sequence alignment of known seed size–related genes indicated that 129 DEGs were related to flax seed size and weight, and they regulated seed size and weight mainly through phytohormone pathways and TFs. In addition, 54 DEGs were identified as seed size–related candidate genes using a DEG-based association study. Candidate DEGs were artificially selected during flax domestication. These results provide a list of useful candidate genes and a genetic basis for flax seed improvement.

## Data availability statement

The datasets presented in this study can be found in online repositories. The names of the repository/repositories and accession number(s) can be found below: https://www.ncbi.nlm.nih.gov/, PRJNA856842.

## Author contributions

HJ, DG, and LX designed the study. YL, LZ, and FX performed morphological and cellular analyses. HJ and DG analyzed the data. HJ, DG, and LX wrote the manuscript. All authors have read and approved the final manuscript. HJ and DG contributed equally to the study.

## Funding

This study was supported by the National Natural Science Foundation of China (31160056, 32060426, and 32270240), the Resource Platform Project of Xinjiang Uygur Autonomous Region of China (PT1808), and the Science and Technology Innovation Project for Doctoral Students of Xinjiang University of China (XJUBSCX-2017017).

## Acknowledgments

We are very grateful to the Chinese Crop Germplasm Resources Information System (CGRIS), the Plant Gene Resource Centre in Canada (PGRC), and the U.S. National Plant Germplasm System (U.S.NPGS) for providing us with germplasm resources. We thank the Dali Economic Crop Research Institute and the YiLi Agricultural Sciences Institute for providing experimental fields and field management.

## Conflict of interest

The authors declare that the research was conducted in the absence of any commercial or financial relationships that could be construed as a potential conflict of interest.

## Publisher’s note

All claims expressed in this article are solely those of the authors and do not necessarily represent those of their affiliated organizations, or those of the publisher, the editors and the reviewers. Any product that may be evaluated in this article, or claim that may be made by its manufacturer, is not guaranteed or endorsed by the publisher.
