# Supplementary material for: RNA-Seq combined with population-level analysis reveals important candidate genes related to seed size in flax (Linum usitatissimum L.)
Source: Front Plant Sci. 2022 Oct 25;13:1015399. doi: 10.3389/fpls.2022.1015399 (PMC9641021; doi:10.3389/fpls.2022.1015399)
Supplement: Supplementary file 1 [file DataSheet_1.pdf]

Supplementary Figures

RNA-Seq combined with population-level analysis reveals important candidate genes related to seed size in flax (*Linum usitatissimum* L.)

Haixia Jiang[†], Dongliang Guo[†], Yuanyuan Liu, Leilei Zhu, Fang Xie, and Liqiong Xie[*]

Xinjiang Key Laboratory of Biological Resources and Genetic Engineering, College of Life Science and Technology, Xinjiang University, Urumqi, Xinjiang, China

[*] Corresponding author: picea@xju.edu.cn (L. Xie).

[†]These authors contributed equally to this work.

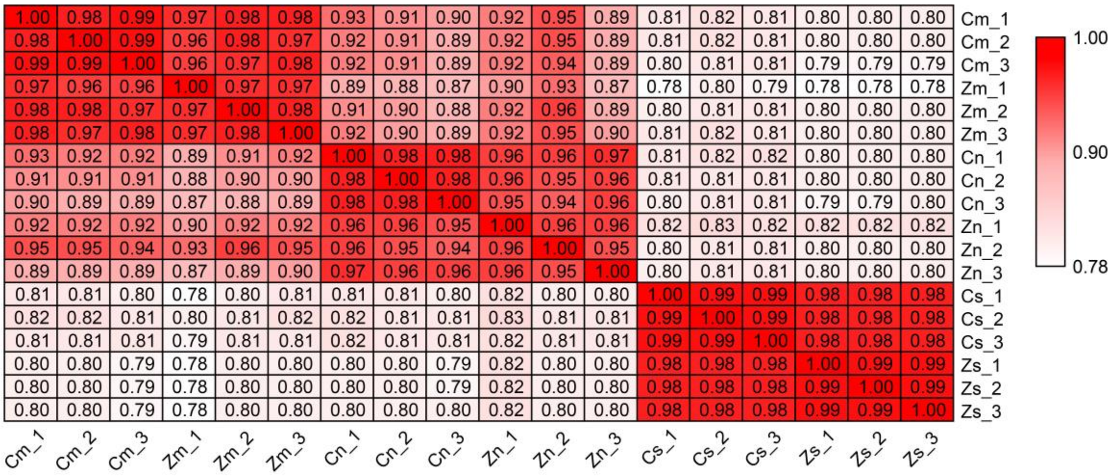

Supplementary Figure S1. Pearson correlation coefficients (*r*) of 18 libraries.

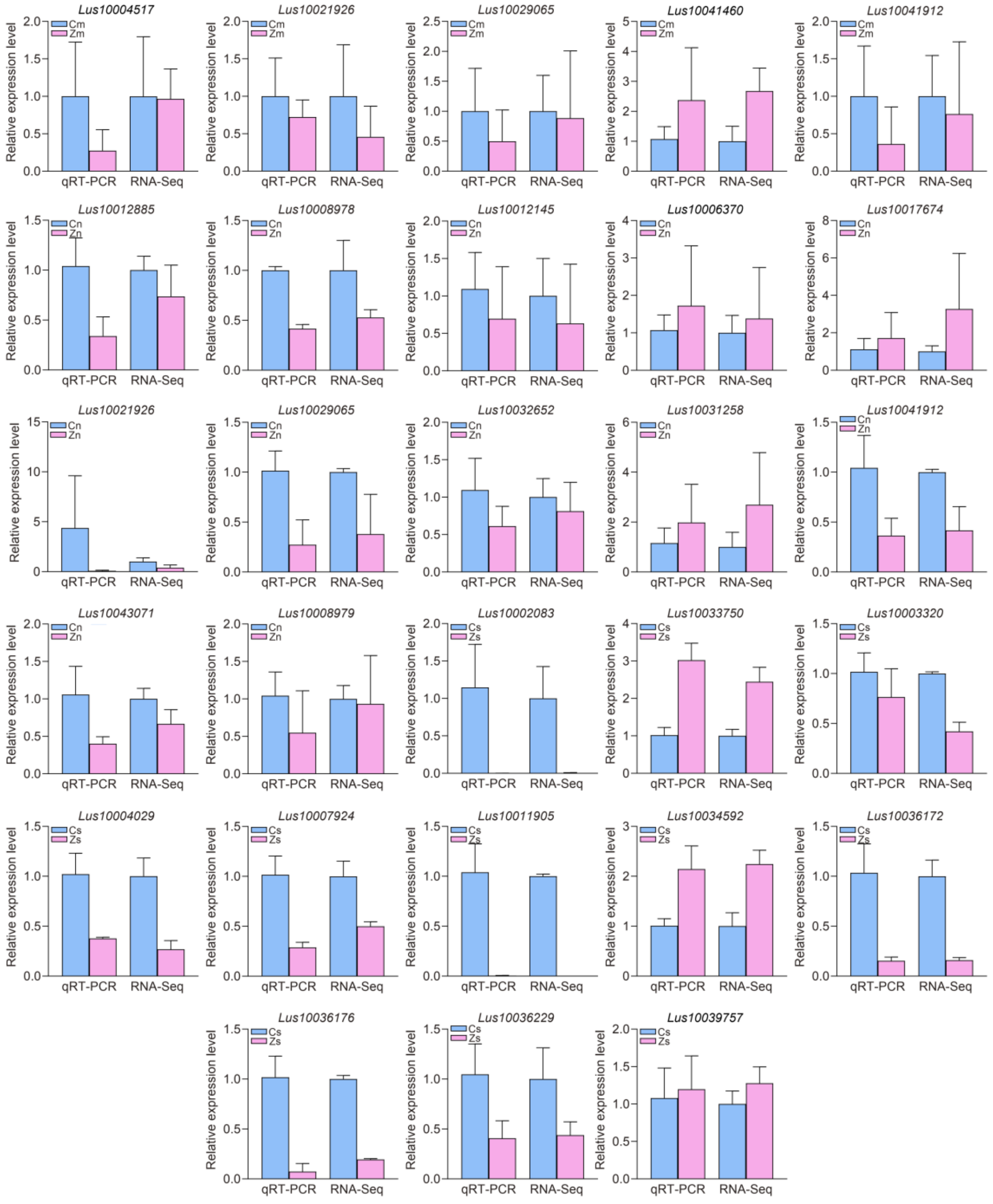

Supplementary Figure S2. Verification of the RNA-seq data in the embryo, endosperm, and seeds of flax by qRT-PCR analysis.

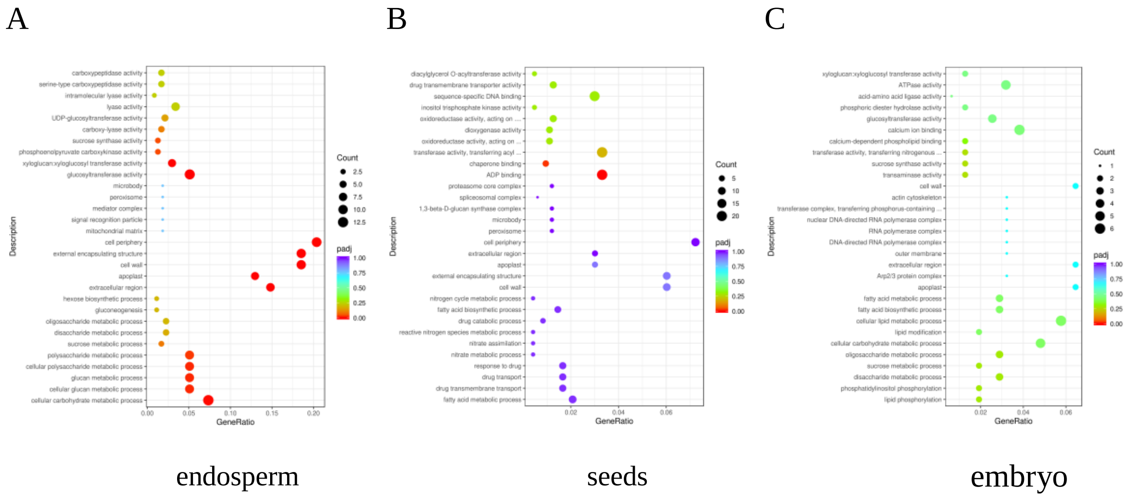

Supplementary Figure S3 Gene ontology (GO) enrichment analysis of DEGs identified in the endosperm, seeds, and embryo. (A) The GO pathway enrichment analysis DEGs identified in the endosperm. (B) The GO pathway enrichment analysis DEGs identified in the seeds. (C) The GO pathway enrichment analysis DEGs identified in the embryo.

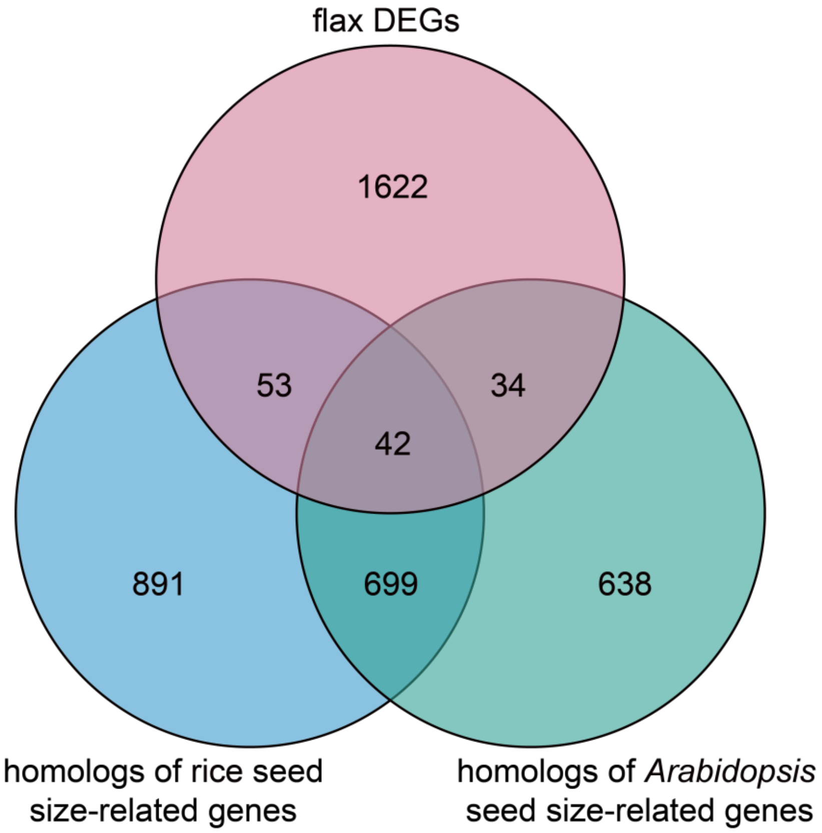

Supplementary Figure S4. Seed size-related genes in model plant *Arabidopsis* and rice were aligned homologous to flax.

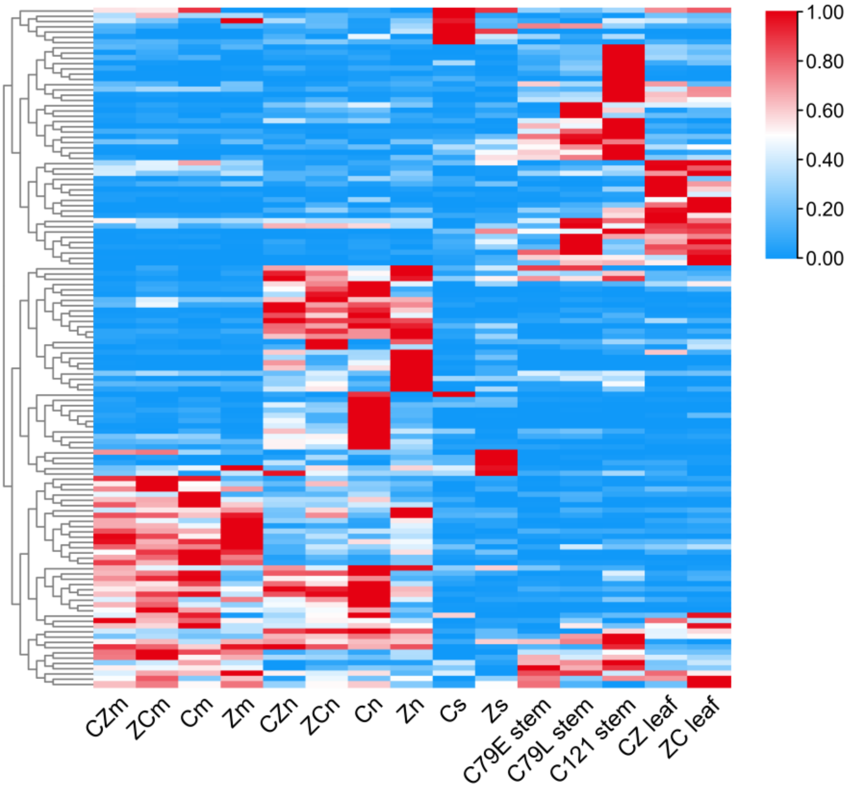

Supplementary Figure S5. Hierarchical clustering and heatmap analysis of the 129 seed size-related DEGs identified in the endosperm, embryo, and seeds by peptide sequence alignment. The FPKM of Cn, Zn, CZn, ZCn, CZm, ZCm, CZ leaves and ZC leaves were obtained from NCBI (PRJNA720521) and the FPKM of stem tissues were obtained from NCBI (PRJNA749598). The FPKM value was normalized by Zero to One method.

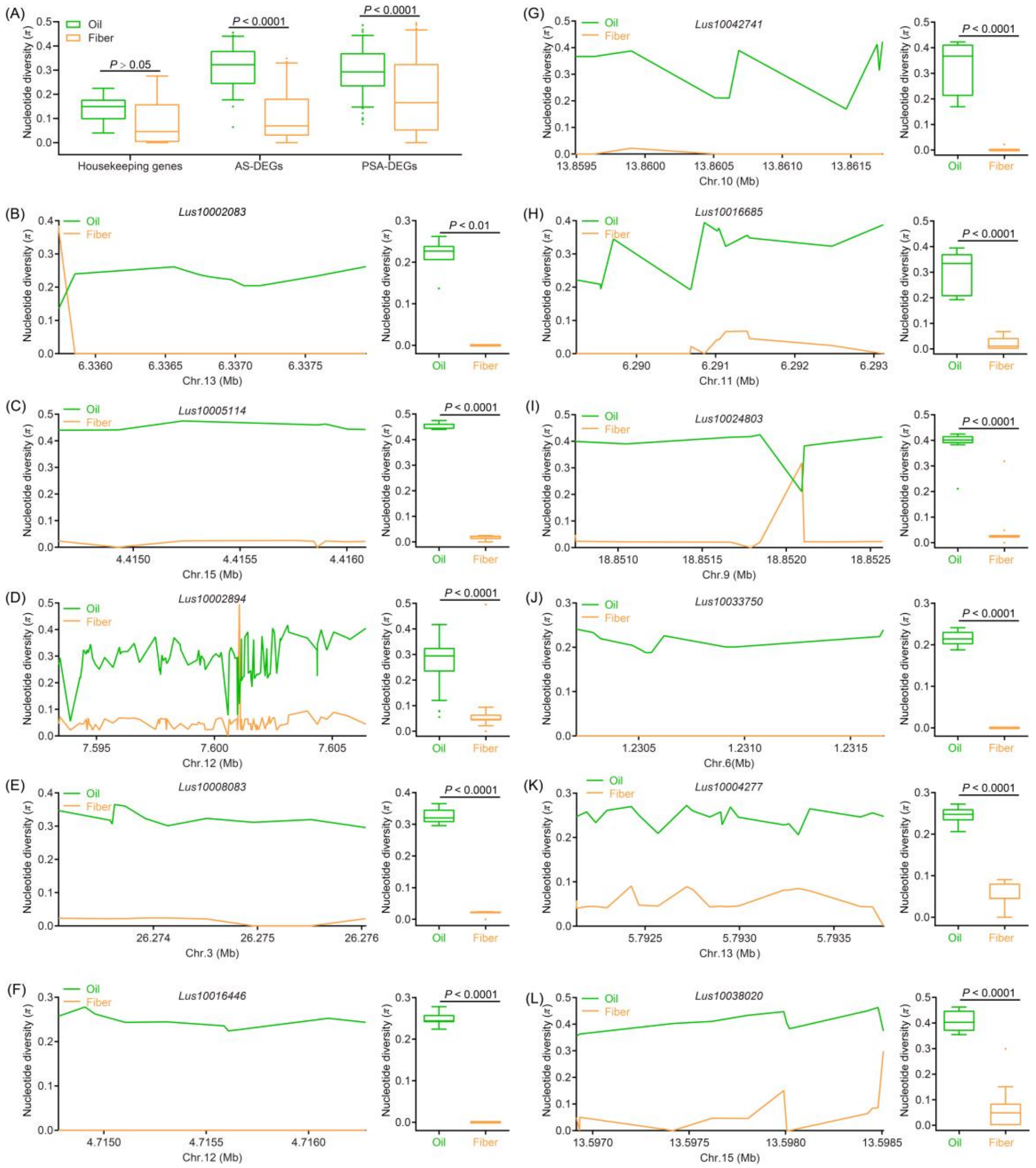

Supplementary Figure S6. The nucleotide diversity distribution of seed size candidate DEGs and housekeeping genes. (A) Boxplots for nucleotide diversity of housekeeping genes, 54 seed size candidate DEGs identified by DEGs-based association study (AS-DEGs), and 129 seed size candidate DEGs obtained using peptide sequence alignment (PSA-DEGs) between oil flax (green) and fiber flax (orange) subgroups. (B-L) The nucleotide diversity distribution of 11 AS-DEGs between oil and fiber flax subgroups. The difference was analyzed by two-tailed *t* tests.

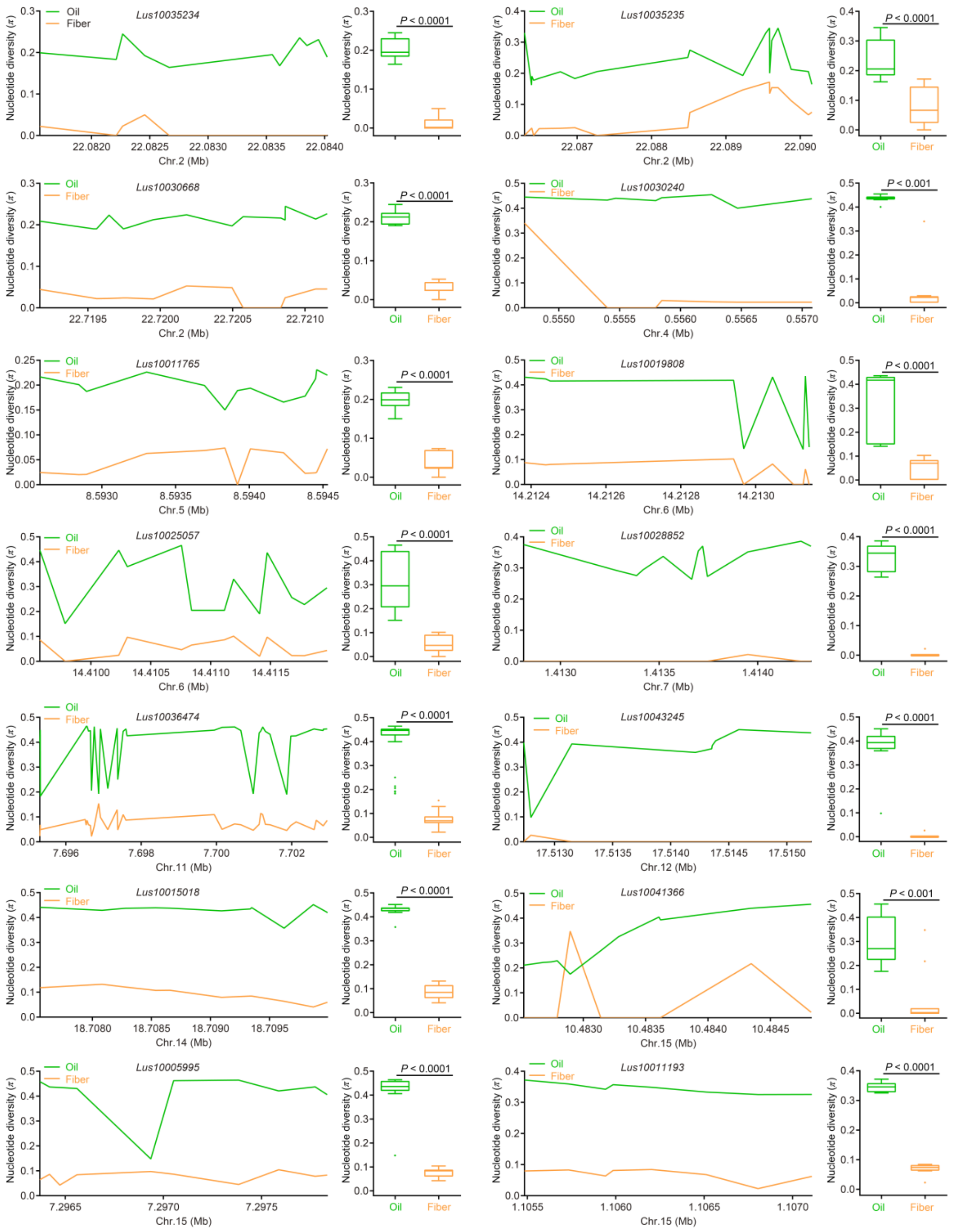

Supplementary Figure S7. The nucleotide diversity distribution of 14 seed size-related DEGs obtained by peptide sequence alignment between oil and fiber flax subgroups. The difference was analyzed by two-tailed *t* tests.
